# G-Protein-Coupled Estrogen Receptor (GPER) in Inflammatory Myopathies

**DOI:** 10.3390/neurolint17070109

**Published:** 2025-07-17

**Authors:** Delia Righi, Diego Lopergolo, Nila Volpi, Daniela Franci, Paola Lorenzoni, Margherita Aglianò, Gianna Berti, Carlo Manco, Nicola De Stefano, Federica Ginanneschi

**Affiliations:** 1 Department of Medical, Surgical and Neurological Sciences, University of Siena, 53100 Siena, Italy; d.righi4@student.unisi.it (D.R.); diego.lopergolo@unifi.it (D.L.); volpi@unisi.it (N.V.); daniela.franci@unisi.it (D.F.); gianna.berti@unisi.it (G.B.); c.manco@student.unisi.it (C.M.);; 2 UOC Neurologia, Azienda Ospedaliera Universitaria Senese, 53100 Siena, Italy

**Keywords:** G protein-coupled estrogen receptor, inflammatory myopathies, skeletal muscle, immunohistochemistry

## Abstract

**Background/Objectives**: Given the multifaceted role of estrogen hormones in skeletal muscle pathophysiology and their well-established immunomodulatory properties, this study aimed to characterize the expression of the G-protein-coupled estrogen receptor (GPER) in patients with inflammatory myopathies (IM). **Methods**: Immunohistochemical analysis was performed on muscle biopsies from 13 patients with IM, 11 with non-inflammatory myopathies (N.IM), and 5control subjects. Intergroup differences in GPER score were statistically evaluated. We performed an analysis based on the Visual Analog Scale (VAS). The scoring system evaluates overall pathology (VAS score) based on four distinct components: inflammation, vascular involvement, myopathic changes, and connective tissue alterations. **Results**: Immunolocalization analysis demonstrated that GPER is constitutively expressed in human skeletal muscle and is upregulated in IM. Enhanced expression included both sarcolemmal and intracellular membrane localization. Notably, GPER upregulation showed a positive correlation with the severity of tissue inflammation. The IM group had significantly higher VAS scores compared to both the N.IM and control groups. **Conclusions**: We provide the first histopathological characterization of GPER expression in human skeletal muscle. In IM, GPER upregulation may play a protective role by negatively modulating the release of inflammatory mediators, as suggested by experimental evidence from other models of inflammation. The emerging therapeutic development of GPER agonists may represent a promising avenue for the treatment of inflammatory myopathies.

## 1. Introduction

Inflammatory myopathies (IM) are a heterogeneous group of rare, acquired muscle diseases characterized by muscle inflammation and weakness, often accompanied by extramuscular manifestations involving the skin, lungs, or joints. Based on clinico-pathological features, several phenotypes characterized by distinct mechanisms of muscle damage have been identified, including dermatomyositis, polymyositis, autoimmune necrotizing myopathy, inclusion body myositis (IBM), and overlap syndromes associated with systemic autoimmune diseases, with the highest prevalence observed in association with antisynthetase syndrome [1].

Estrogen hormones regulate biological processes in tissues across multiple systems beyond the reproductive organs, modulating both metabolic and immune responses. The immunomodulatory role of estrogens is well established in various autoimmune diseases, exhibiting either proinflammatory effects, as seen in systemic lupus erythematosus and rheumatoid arthritis, or anti-inflammatory properties, as observed in conditions such as nephritis and neuroinflammation [2].

The myocardium and skeletal muscle are targets of estrogen hormones, which modulate glucose and lipid homeostasis, mitochondrial function and turnover, as well as mechanisms involved in oxidative stress [3,4,5]. Moreover, experimental studies have demonstrated that estrogens contribute to the regenerative capacity of muscle following inflammatory injury or other damaging events, such as intense exercise-induced stress [6]. After muscle damage, estrogens induce the activation of satellite cells, which proliferate and differentiate into active myoblasts contributing to myofiber regeneration [7,8], while also suppressing the leukocytic inflammatory response.

Estrogen exerts its biological effects via both genomic and non-genomic pathways. The genomic pathway involves estrogen binding to ERα and ERβ receptors, which translocate to the nucleus, bind to estrogen response elements, and regulate gene transcription. Non-genomic effects involve estrogen binding to the G-protein-coupled estrogen receptor (GPER). GPER is localized both on the plasma membrane and within intracellular membrane compartments, including the endoplasmic reticulum and Golgi apparatus. GPER can move intracellularly and is mainly responsible for rapid, non-genomic responses, acting as a transcriptional regulator through second messenger signaling pathways [9,10].

Estrogen receptors are expressed in peripheral B and T lymphocytes, as well as in monocytes, eosinophils, and neutrophils, thereby modulating the immune response [11,12]. ERα primarily exerts its functions in the mammary gland, uterus, and metabolic processes, whereas ERβ predominantly acts within the nervous and immune systems [13]. GPER, a seven-transmembrane G protein-coupled receptor, was identified as an orphan receptor in the late 1990s [14]. It binds 17β-estradiol and activates downstream signaling pathways [15]. Under physiological conditions, GPER is expressed in various tissues, including highly metabolic tissues such as muscle and adipose tissue, as well as endothelial and vascular smooth muscle cells, where it helps regulate vascular tone and blood pressure [16]. GPER is also implicated in several tumor types [17] and is present in inflammatory cells. Accordingly, its immune-related roles have been widely studied in inflammatory and immune-mediated diseases.

Regarding the skeletal muscle pathophysiology, animal studies indicate that ERβ regulates muscle regeneration after injury or inflammation by stimulating anabolic pathways, activating satellite cells, and modulating immune responses [18]. Neutrophil infiltration in inflamed muscle increases after ovariectomy and decreases with estrogen treatment, while ER antagonists reduce estrogen’s effects on leukocyte infiltration [19]. In the dystrophinopathy (mdx) mouse model, ERα and ERβ expression is upregulated [20]. Several muscle pathologies have been partially linked to decreased estrogen levels [21]. Concerning IM, a relationship has been established between the use of aromatase inhibitors—enzymes that convert androgens to estrogens—and the new onset of IM [22].

However, to date, studies investigating the association between GPER expression and skeletal muscle disorders remain lacking [23]. Furthermore, despite the complex biological effects of estrogens on skeletal muscle, data on the precise localization of different estrogen receptor types in human muscle tissue are limited. Only a few immunohistochemical studies have addressed ERα and ERβ expression in muscle [12,24], and no studies to date have examined the membrane receptor GPER. The present study aims to assess the expression pattern of GPER in muscle samples from subjects with IM and non-inflammatory myopathies (N.IM).

## 2. Materials and Methods

### 2.1. Patients

The study was conducted on human skeletal muscle samples obtained from muscle biopsies performed for diagnostic purposes on patients followed by the Neurology Unit of the University Hospital of Siena. Our cohort consisted of 29 subjects. Specifically, we included 13 patients with IM, diagnosed according to current clinicopathological criteria [25]. Among them, the diagnoses were as follows: inclusion body myositis (IBM), 5 patients; overlap myositis, 3; non-specific myositis, 1; myositis with mitochondrial damage, 2; necrotizing autoimmune myopathy, 1; and dermatomyositis, 1. Eleven patients were classified as N.IM. Five subjects were enrolled as controls, whose biopsy specimens were taken due to clinical suspicion but showed no pathological changes. All subjects underwent a vastus lateralis muscle biopsy. Muscle fragments obtained from the biopsy were rapidly frozen in liquid nitrogen-cooled isopentane and stored at −80 °C until use.

All experiments described were approved by the local ethical committee (Regional Ethics Committee for Clinical Trials of the Tuscany Region, n. 17397, approval date 20 July 2020). All procedures were conducted in accordance with the Helsinki Declaration of 1975. Muscle biopsies were performed with the written informed consent of patients.

### 2.2. Immunohistopathological Staining

Cryostatic sections, 7 µm thick, mounted on silanized glass slides (SuperFrostR Plus, Menzel GmbH & Co., Bielefeld, Germany) were subjected to immunoperoxidase staining using a GPER primary antibody (1:250; Invitrogen, Thermo Fisher Scientific, Lenexa, KS, USA) with the IHC Select Immunoperoxidase Secondary Detection System Kit (MERK, Millipore, Darmstadt, Germany), based on the streptavidin-biotin-peroxidase method. The reaction was visualized with a solution of 3,3′-diaminobenzidine and urea (3,3′-DAB, SigmaFast, Sigma-Aldrich, Merk, Darmstadt, Germany 1 mg). Omission of the primary antibody was performed as a negative control.

### 2.3. Semi-Quantitative Analysis of Immunohistochemical Slides

The entire histological slide was scanned using the NanoZoomer Digital Slide Scanner (S360, Hamamatsu, Japan). Subsequently, semi-quantitative analysis was performed with QuPath, an open-source bioimage analysis software Version 0.6.0 developed with JavaFX, specifically designed for digital pathology [26]. The analysis was based on the Visual Analog Scale (VAS) score, a method previously described by Preuße et al. [27]. This scoring system evaluates the overall pathology observed in cryostat sections of skeletal muscle by assessing four distinct components: inflammatory, vascular, myopathic changes, and connective tissue alterations. In the evaluation of inflammation, particular attention was given to the abundance of inflammatory cells.

Regarding myopathic changes, our evaluation includes the presence of damaged fibers, atrophic fibers, and centrally located nuclei. In the vascular domain, we assess alterations in vessel wall integrity and the loss of vessels. Finally, we evaluate potential increases in endomysial and perimysial connective tissue. VAS scores reflect the overall abnormality of the muscle but do not correspond to a simple sum of the four individual components. Scores range from 0 to 10, with higher values indicating greater muscle abnormality. GPER expression density was quantified by calculating the ratio of GPER-positive fibers to the total number of fibers counted within a randomly selected area of 706,583 µm^2^. A semi-quantitative score was assigned based on the percentage of reactive fibers as follows: 0 for no staining, 1 for up to 3% reactive fibers, 2 for 3–5%, 3 for 6–8%, and 4 for more than 8% reactive fibers. This scoring method was consistently applied across all sections and patients. Each selected area contained at least 150 muscle fibers for analysis.

### 2.4. Statistical Analysis

Statistical analysis was performed by Jamovi SoftwareVersion 2.6.26 for Windows. GPER values and VAS scores were treated as ordinal variables and analyzed as dependent variables. Ordinal logistic regression was performed to assess the effects of age and group on these dependent variables. The independent variable ‘group’ was categorized into three levels: IM, N.IM, and controls. Model fit was evaluated using standard goodness-of-fit indices, including Cox &Snell’s R^2^ (R^2cs^), Nagelkerke’s R^2^(R^2n^), and the Akaike Information Criterion. The Kruskal–Wallis test was used to compare continuous variables among the three groups. The Dunn test was used for multiple comparison. The χ^2^ test was employed to analyze differences in sex among the three groups. Quantitative data were analyzed using two-tailed Spearman’s rank correlation to assess the strength of associations between variables, with age included as a covariate. The Mann–Whitney U test was used to compare data between two groups (IM and N.IM).

Finally, we conducted a power analysis to determine whether our sample sizes (13 subjects in the IM group and 11 subjects in the N.IM group) were sufficient to yield reliable statistical results for GPER and VAS scores between the two groups.

Statistical significance was set at *p* < 0.05.

## 3. Results

Of the 29 enrolled subjects, 13 had IM (median age 68.5 years, 8 males), 11 had N.IM (median age 61.5, 7 males), and 5 were controls (median age 64 years, 2 males). No statistically significant differences in age and sexes were observed among patients across the three groups (*p* = 0.25 and *p* = 0.54 respectively). In Table 1, histopathologic and clinical parameters across subject groups were described.

**Figure 1:**
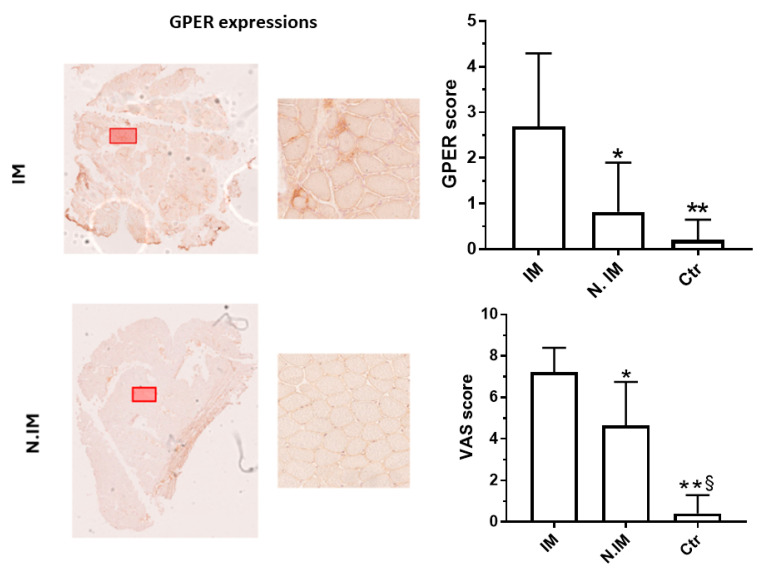
On the left, full muscle biopsy sections from inflammatory myopathy (IM) and non-inflammatory myopathy (N.IM) cases, demonstrating differential expression of GPER. The red boxes highlight areas with higher muscle fibers density. These areas are magnified in the adjacent panels. In the upper-right panel, histograms display the GPER scores in IM, N.IM, and control subjects (Ctr). Significant differences are indicated by asterisks. IM vs. N.IM: *p* = 0.023 (*). IM vs. Ctr: *p* = 0.011 (**). In the lower-right panel, histograms display the VAS scores in IM, N.IM, and Ctr. Significant differences are indicated by asterisks. IM vs. N.IM: *p* = 0.0004 (*); IM vs. Ctr: *p* < 0.0001 (**). N.IM vs. Ctr: *p* < 0.0001 (§).

GPER scores were significantly different among the three groups (*p* = 0.003). Specifically, GPER expression was higher in the IM group compared to both the N.IM (*p* = 0.011) and control groups (*p* = 0.023). No significant differences were observed in GPER expression between the N.IM and control groups (see upper-right panel in the Figure 1).

Considering only IM and N.IM groups, the mean GPER scores were 2.6 (SD = 1.6) for the IM group and 0.82 (SD = 1.1) for the N.IM group. Using these values, we calculated a Cohen’s d effect size of approximately 1.19, indicating a large effect. Assuming a two-tailed independent samples *t*-test with a significance level of α = 0.05, the achieved power with the current sample sizes (number = 13 for IM and number = 11 for N.IM) is estimated to be between 80% and 90%. This indicates that the study has sufficient power to detect the observed large difference in clinical scores between the two groups.

VAS scores also showed significant differences among the three groups (*p* < 0.0001). The IM group had significantly higher VAS scores compared to both the N.IM (*p* = 0.0004) and control groups (*p* < 0.0001). Additionally, the control group showed significantly higher VAS scores than the N.IM group (*p* < 0.0001) (see lower-right panel in the Figure 1).

Considering only IM and N.IM groups, the mean VAS scores were 7.38 (SD = 1.2) and 4.64 (SD = 2.1), respectively. Assuming a two-tailed independent samples *t*-test with a significance level of α = 0.05, the probability that a randomly selected score from IM group exceeds a randomly selected score from N.IM group was estimated to be 0.74, corresponding to a medium-to-large effect size (approximate Cohen’s d ≈ 0.9). Given the sample sizes and effect size, the estimated statistical power of the Mann–Whitney U test is approximately 75–80%.

**Figure 2:**
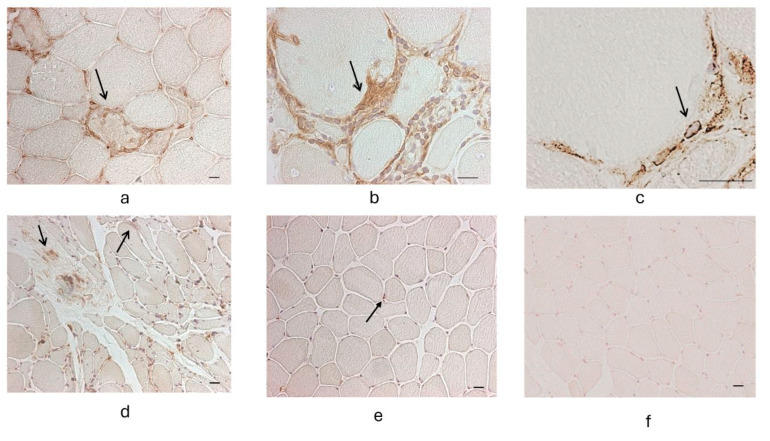
Panels (**a**–**c**): cases of inclusion body myositis (IBM). Panel (**d**): case of myositis, non-IBM. Panel (**e**): case of non-inflammatory myopathy. Panel (**f**): negative control. Immunoperoxidase staining for GPER, resulting in the darker brown areas evident in panels (**a**–**e**). It is evident an upregulated GPER reactivity, not only in inflammatory cells but also on the sarcolemma and within degenerating fibers (arrows), particularly adjacent to inflammatory infiltrates. Sarcolemmal expression of GPER is very weak in cases with myopathic damage but without prominent inflammation, though it remains detectable in endomysial cells (arrow in panel **e**). Scale bar: 50 µm (panels **a**,**d**,**e**,**f**), 100 µm (panel **b**), 250 µm (panel **c**).

Immunohistological localization of GPER in normal skeletal muscle samples revealed no immunoreactivity. In N.IM, GPER immunoreactivity was more pronounced, particularly in degenerating and regenerating fibers. In IM, an upregulation of GPER was observed on the sarcolemma and especially around inflammatory infiltrates (Figure 2).

Correlation analysis revealed significant positive correlations in both IM and N.IM cohorts between the GPER score and the VAS score (*p* = 0.008, Spearman’s rho = 0.56), as well as between the GPER score and the inflammation score (*p* = 0.009, Spearman’s rho = 0.55). Additionally, the GPER score showed significant correlations with the myopathic changes score (*p* < 0.001, Spearman’s rho = 0.69) and the connective tissue changes score (*p* = 0.005, Spearman’s rho = 0.61). No significant correlation was observed between the GPER score and vascular changes (*p* = 0.51, Spearman’s rho = 0.16).

The ordinal logistic regression models were both statistically significant (GPER X2 (3) = 17.9, *p* < 0.001; VASX2 (3) = 33.2, *p* < 0.01). The proportion of variance explained ranged from 13% to 30% for the GPER model (R^2cs^ = 0.13, R2n = 0.30, AIC = 69.5) and from 13% to 35% for the VAS model (R^2cs^ = 0.13, R2n = 0.35, AIC = 97.6). Regarding the model coefficients, group membership had a statistically significant effect in both models (*p* < 0.001), whereas age was not a significant predictor (GPER: *p* = 0.65; VAS: *p* = 0.33).

In a subgroup analysis, we compared GPER scores between patients with IBM and those with inflammatory myositis non-IBM: the mean GPER score was 3.28 (SD = 1.2) in IBM patients and 2.00 (SD = 1.79) in patients with inflammatory myositis non-IBM. Statistical comparison using Mann–Whitney U test showed no significant difference between groups (*p* = 0.18).

Finally, considering all patients with IM and N.IM, we investigated possible differences in GPER scores between males and females. TheMann–Whitney U test showed no significant difference between groups (*p* = 0.19)(mean 2.38, SD = 1.5 in males and mean 1.45, SD = 1.7 in females).

## 4. Discussion

In the present study, we observed an increased sarcolemmal and intracellular expression of GPER in the IM group compared to the N.IM and control groups. Furthermore, our findings reveal a significant correlation between GPER expression and the overall VAS score. Notably, inflammation, myopathic changes, and connective tissue involvement demonstrated a stronger association with GPER expression. []These correlations further support the hypothesis that GPER may be functionally implicated in the inflammatory and structural remodeling processes characteristic of IMs.

The observed GPER overexpression in muscle fibers affected by the inflammatory process may suggest a potential role for GPER in modulating inflammatory responses [28]. Consistent with our findings, increased GPER expression has also been reported in Crohn’s disease, where it shows membrane localization in mucin-producing cells and strong cytoplasmic expression in enterocytes [29]. Interestingly, GPER is expressed in various immune cells, including T cells, B cells, macrophages, eosinophils, and neutrophils, where it contributes to the modulation of the immune microenvironment [12]. In T lymphocytes, GPER expression influences thymic function and promotes IL-10 production in CD4^+^ T cells—particularly in T helper 17 cells—through the activation of the ERK signaling pathway. Additionally, GPER enhances the expression of the transcriptional regulator Foxp3 and upregulates programmed cell death protein 1 (PD-1) in regulatory T cells (Tregs) [12].

The potential protective effects of GPER signaling have also been demonstrated in the central nervous system. GPER activation stimulates the IP3/CaMK signaling pathway, leading to the activation of antioxidant response elements. Additionally, GPER activation promotes Src phosphorylation, which in turn triggers the RAF/RAS/MEK cascade and subsequent activation of the ERK1/2 signaling pathway. This signaling cascade enhances the expression of anti-apoptotic proteins such as Bcl-2, thereby inhibiting neuronal cell death, conferring neuroprotective effects, and mitigating oxidative stress [30].

Consistent with these findings, the involvement of GPER in ischemic stroke has been documented [31]. An upregulation of GPER expression at the microglial level has been observed in murine models of neuroinflammation following ischemic stroke. This increase is interpreted as part of a neuroprotective response, exerting anti-inflammatory effects that contribute to the reduction of ischemic damage [32,33].

Further support for the anti-inflammatory role of GPER comes from evidence indicating its ability to inhibit the NF-κB signaling pathway. This occurs through downregulation of Toll-like receptor 4 expression in lymphocytes and a consequent reduction in the release of pro-inflammatory cytokines, including TNF-α, IL-1β, and IL-6 [34].

Consequently, the observed GPER overexpression in our IM group may represent an anti-inflammatory response to the ongoing myositic process. Inflammatory myopathies are characterized by the infiltration of T cells into muscle tissue, however, the mechanisms underlying T-cell recruitment and persistence within muscle remain poorly understood [35]. A deeper understanding of the pathways driving T-cell accumulation in muscle would be essential for the development of novel therapeutic strategies for patients with IM.

No association was found between the vascular score—which evaluates the integrity and loss of blood vessels—and GPER expression. This suggests that while GPER may contribute to inflammatory and myopathic tissue changes, its expression is not directly linked to microvascular structural alterations in muscle tissue. Indeed, GPER plays a significant role in the regulation of vascular tone [16]. Its activation by endogenous estrogens or selective agonists has been shown to induce vasodilation through both endothelium-dependent and -independent mechanisms [36]. One of the primary pathways involves the stimulation of nitric oxide production via endothelial nitric oxide synthase leading to the relaxation of vascular smooth muscle [37]. Additionally, GPER activation can modulate intracellular calcium signaling and reduce vasoconstrictor responses, contributing further to the maintenance of vascular homeostasis [38]. These effects underscore the potential of GPER as a vasoprotective target in vascular dysfunction and inflammatory diseases.

Based on our findings, no significant sex-related differences in muscle GPER expression were observed. Specifically, when analyzing all patients (IM and N.IM) GPER scores did not differ significantly between males and females. These results suggest that GPER expression in skeletal muscle is not influenced by sex, at least within the context of the disease states examined in this study.

While no previous data specifically address sex-related differences in GPER expression in skeletal muscle, findings from other tissues have been inconclusive and sometimes contradictory. Some studies suggest that GPER expression patterns may vary according to age, species, sex, and tissue type. For instance, GPER mRNA expression in skeletal muscle has been reported to be higher in premenopausal women compared to postmenopausal women, although this difference did not reach statistical significance [39]. In mouse skeletal muscle, GPER mRNA levels are nearly fourfold higher in females compared to males [40]. Moreover, GPER mRNA expression is higher in the female soleus muscle compared to the extensor digitorum longus muscle [41], suggesting greater expression in slow-twitch muscle fibers.

However, GPER activation—driven by anti-inflammatory signaling pathways and the established involvement of satellite cells in muscle regeneration—may exert a dominant influence that overshadows baseline sex-related differences in GPER expression. In inflammatory conditions such as myopathies, the local microenvironment is characterized by a complex interplay of immune responses and tissue repair mechanisms. These processes likely enhance GPER activity regardless of the patient’s sex, thereby minimizing or neutralizing the physiological expression differences typically observed between males and females under non-pathological conditions. This could explain the absence of significant sex-based differences in GPER scores in our cohort.

In the present study, a subclass of (IM) is represented by IBM, the most common IM affecting men over 50 years of age and characteristically resistant to immunosuppressive therapy. The GPER score in patients with IBM was comparable to that observed in other forms of inflammatory myopathies, in which inflammation represents the predominant pathological feature. The pathophysiology of IBM involves degenerative, inflammatory, and mitochondrial alterations. The phenotype is marked by endomysial inflammation, autophagic vacuoles, amyloid deposits, and cytochrome c oxidase (COX)-deficient fibers, with mitochondrial paracrystalline inclusions observable by transmission electron microscopy [42]. GPER expression appears to play a role in several signaling pathways, including mitochondrial function, acting as a regulator of cellular energy homeostasis and metabolism [43]. This may be linked to estrogen’s ability to suppress uncoupling protein 3, which contributes to proton leakage and reduced energy production in mitochondria of skeletal muscle fibers [44]. Given that therapeutic targeting of GPER is currently under preclinical investigation for metabolic disorders and potentially cancer—using the small-molecule GPER agonist G-1 (Tespria) [45,46]—GPER could represent a promising novel target for IBM treatment. According to our data, in a mouse model, the use of GPER agonist [45] reversed the inflammatory phenotype and reduced plasma concentrations and expression of circulating and tissue inflammatory cytokines. Moreover, evidence for the anti-inflammatory effects of GPER agonist derives from reports of experimental autoimmune encephalomyelitis where G-1 was able to reduce the disease severity [47].

## 5. Conclusions

In conclusion, our results provide the first histopathological characterization of GPER expression in human skeletal muscle. We demonstrate increased sarcolemmal and intracellular expression of GPER in the context of muscle inflammation. Although these findings require validation in larger cohorts, they are supported by experimental evidence of GPER upregulation in inflammatory diseases across various tissues. The emerging therapeutic development of GPER agonists may represent a promising avenue for the treatment of inflammatory myopathies. Although direct clinical studies on GPER agonists are currently limited, preclinical data in related inflammatory myopathies and muscle injury models support the rationale for exploring GPER-targeted therapies. By modulating the muscle microenvironment to favor repair and reduce inflammation, GPER agonists have the potential to improve muscle function and slow disease progression.

## Figures and Tables

**Table 1:** Histopathologic and clinical parameters across subject groups.

Subject	Group	Histopathologic Findings	GPER Medium Score	GPER Score	Inflammation Score	Myopathic Changes Score	Vascular Changes Score	Connective Tissue Changes Score	VAS Score
1	IM	Inflammatory Myopathy	GPER/TOT: 3.85% AF/NAF: 1.78%; RAF/AF: 100%	2	2	1	0	0	7
2	IM	Inflammatory Myopathy	GPER/TOT: 12.03% AF/NAF: 34.2% RAF/AF: 19.30	4	2	2	1	2	9
3	IM	IBM	GPER/TOT: 3.2% AF/NAF: 1.91% RAF/AF: 66%	2	0	1	1	1	5
4	IM	Inflammatory Myopathy	GPER/TOT: 7.1% AF/NAF: 7% RAF/AF: 49.19%	4	2	2	0	1	8
5	IN	IBM	GPER/TOT: 14.79% AF/NAF: 15.84% RAF/AF: 26.06%	4	2	2	1	1	8
6	IM	IBM	GPER/TOT: 9.5% AF/NAF: 14.24% RAF/AF: 31.37%	4	2	2	1	1	8
7	IM	Dermatomyositis	GPER/TOT: 1.92%	0	1	1	2	0	6
8	IM	IBM	GPER/TOT: 2.44% AF/NAF: 4.26	1	1	2	0	1	6
9	IM	IBM	GPER/TOT: 8.76% AF/NAF: 7.73% RAF/AF: 31.53%	4	2	2	1	1	8
10	IM	Inflammatory Myopathy	GPER/TOT: 1.72% AF/NAF: 3.51%	0	2	2	1	1	8
11	IM	Inflammatory Myopathy	GPER/TOT: 3.8% FA/FNA: 2.96% RAF/AF: 66.66%	2	2	2	1	1	8
12	IM	IBM	GPER/TOT: 9.02% AF/NAF: 6.29% RAF/AF: 61.9%	4	2	2	1	1	7
13	IM	IBM	GPER/TOT: 9.29% AF/NAF: 16.75% RAF/AF: 17.76%	4	0	2	1	1	6
14	N. IM	Neurogenic atrophy	GPER/TOT: 6.05% AF/NAF: 3.03% RAF/AF: 65%	3	1	2	0	1	8
15	N.IM	Mild myopathic changes	GPER/TOT: 1.61% AF/NAF: 0.79%	0	0	1	1	0	5
16	N.IM	Mild myopathic changes	GPER/TOT: 0.73%	0	0	1	0	0	3
17	N.IM	Mild myopathic changes	GPER/TOT: 2.04%	1	0	1	0	0	2
18	N.IM	Mild myopathic changes	GPER/TOT: 1.73% AF/NAF: 0.73%	0	0	1	0	1	4
19	N.IM	Mild myopathic changes	GPER/TOT: 1.96% AF/NAF: 1.51% RAF/AF: 70.83%	0	0	1.5	0	0	3
20	N.IM	Core myopathy	GPER/TOT: 4.35% AF/NAF: 3.07% RAF/AF: 58.57%	2	0	2	0	1.5	7
21	N.IM	Mild myopathic changes	GPER/TOT: 3.78% AF/NAF: 4.46%	2	1	1.5	1	1	6
22	N.IM	HIV-related myopathy	GPER/TOT: 2.19% AF/NAF: 1.69%	1	0	1	0	0	2
23	N.IM	SYNE1-related myopathy	GPER/TOT: 0.45%;	0	0				7
24	N.IM	Neurogenic changes	GPER/TOT: 0.45%;	0	2	1	0	1	4
25	Ctr	No pathological changes	No evidence of GPER-reactive fibers or atrophy	0	0	0	0	0	0
26	Ctr	Sarcoidosis	No evidence of GPER-reactive fibers or atrophy	0	0	0	0	1	2
27	Ctr	No pathological changes	No evidence of GPER-reactive fibers or atrophy	0	0	0	0	0	0
28	Ctr	No pathological changes	No evidence of GPER-reactive fibers or atrophy.	0	0	0	0	0	0
29	Ctr	Sarcoidosis	GPER/TOT: 0.52%	1	0	0	0	0	0

Connective Tissue Score: Assesses the increase in endomysial and perimysial connective tissue. Scores range from 0 to 2. GPER Score: Measures the density of GPER-reactive fibers. Scores range from 0 to 4. Inflammatory Score: Assesses the abundance of inflammatory cells in muscle tissue. Scores range from 0 to 2. Myopathic Score: Evaluates the presence of damaged fibers, atrophic fibers, and centrally located nuclei. Scores range from 0 to 2. VAS Score: A visual analogue scale used to assess overall muscle pathology. Scores range from 0 to 10. Vascular Score: Examines the integrity and loss of blood vessels. Scores range from 0 to 2. GPER/TOT: Represents the proportion of GPER-reactive fibers relative to the total muscle fiber count. AF/NAF: Ratio of atrophic fibers to non-atrophic fibers. RAF/AF: Ratio of GPER-reactive atrophic fibers to total atrophic fiber. If this ratio is not reported, it indicates the absence of either atrophic fibers or GPER-reactive atrophic fibers. Ctr: control subjects; IBM: inclusion body myopathy; IM: inflammatory myopathy; N.IM: non-inflammatory myopathy.

## Data Availability

The original contributions presented in this study are included in the article. Further inquiries can be directed to the corresponding author.

## Abbreviations

The following abbreviations are used in this manuscript:

GPER	G-protein-coupled Estrogen Receptor
IM	Inflammatory Myopathy
N.IM	Non-inflammatory Myopathy
VAS	Visual Analog Scale
